# Coexistence of critical phenomena: the concept of manifold multi-spectral criticality

**DOI:** 10.1038/s41598-024-53014-2

**Published:** 2024-03-21

**Authors:** Michał Chorowski, Ryszard Kutner, Zbigniew R. Struzik

**Affiliations:** 1 Faculty of Physics, University of Warsaw, Pasteur Str. 5, 02093 Warsaw, Poland; 2 Graduate School of Education, The University of Tokyo, 7-3-1 Hongo, Bunkyo-ku, Tokyo, 113-0033 Japan

**Keywords:** Phase transitions and critical phenomena, Computational models, Computational science

## Abstract

Prompted by the ubiquity of empirical observations of critical phenomena, often in non-equilibrium macrostates, we developed a modelling approach in which several critical phenomena coexist. Instead of a single critical point, many coexisting critical points in the system are identified, forming a one-dimensional critical manifold. Identified within our game-of-life-like heterogeneous agent-based simulation model, where agents can be created and annihilated in the presence of a catalyst, each critical point belonging to the critical manifold is associated with a multi-spectrum of critical exponents. We find this situation in non-equilibrium mixed percolation-like macrostates obeying continuous phase transitions. These macrostates are quasi-stationary, where some system characteristics are time-independent while others are not. This novel look at universality signals the existance of complexity of critical phenomena richer than described to date.

## Introduction

The primary motivation of this work is the everyday observation that a broad range of different critical phenomena can coexist. A necessary condition for this is the existence in the system of many critical points available for the system concerned. The system can reach these critical points following multiple independent trajectories in the multivariable system’s phase space. This paradigm is a reminiscence of natural systems’ ease of achieving critical macrostates. More often than not, this goes together with the observation that the system can simultaneously exhibit stationary but non-equilibrium in the statistical physics sense^1^) and non-stationary characteristics. We will refer to such a macrostate of the system as quasi-stationary. Indeed, this work aims to model both the situation just described.

The phenomenon of criticality has been intensively studied in agent-based modelling (ABM), physics of phase transitions, and, more generally, in the science of complexity (SC)^2–13^. In physics, a multicritical behaviour has been explored for a considerable time^14–27^. Three different canonical examples are indicated below. (i) A straightforward example could be material possessing weak ferromagnetism in a critical region subjected to hydrostatic pressure^28^. Notably, the origin of the weak ferromagnetic moment is related to two opposing sub-lattices slightly canted concerning each other. Then the Curie/critical temperature is a critical curve, not an isolated point, depending on the pressure and terminating in the multicritical point. (ii) Another compelling example is a uniaxially anisotropic ferromagnet with longitudinal random field applied^20^. In this case—where is worth noting that both in our situation and there involved an exogenous random field—a new multicritical point was found as the intersection of three multicritical lines bicritical, tricritical, and critical end-points. (iii) Further extensive work on the topic^29^ found the global phase diagram of the uniaxially anisotropic ferromagnet, which seems rather exotic. Thanks to this, the authors obtained a deep insight into the nature of the multicritical point, occurring in the five-dimensional space of the uniform longitudinal field, longitudinal random field component, transverse random field component, temperature, and degree of anisotropy. Indeed, applying an ordering field along the easy axis of magnetisation splits it into two symmetrical lines of unique bicritical points. Simultaneously the pure transverse phase of the three-dimensional space (local uniaxial random magnetic field, temperature, degree of anisotropy) spreads into two symmetrical horns. The new multicritical point is of fifth order at this stage. A particular feature of it is the decoupling of the longitudinal and transverse degrees of freedom, which holds for the associated curves of unique bicritical points. Due to the presence of random fields, the results are expected to be correct only in the vicinity of six dimensions. (iv) It is also worth noting an example characteristic for disordered systems, i.e., the existence of a multicritical point in the spin glass^30^. Generally, in the multicritical situation, the curves leading to the multicritical point are the borders of phase coexistence, and they can arise only for more than two phases^23^.

Here we present two related issues—the first showing how our results relate to the well-known phenomenon of multicriticality. Secondly, our scenario plays out not in statistical/thermodynamic equilibrium but in quasi-stationary macrostates. To roughly illustrate our approach, we consider a game-of-life model of heterogeneous agents competing for survival in an exogenous catalytic random field. Agent models are increasingly explored in the context of complexity and criticality. Recent work such as by Ferreira et al^31^ identified during our manuscript’s revision addresses the context of human interactions under global leadership. While interesting and relevant, the model does not account for the evolution of the leader—which is the situation our work addresses.

While such an extended game-of-life model can have multiple applications, we propose a company market interpretation for the model. The content of our work is presented in the ‘Manifold multi-spectral critical phenomena: results and discussion’ section – concentrating on the results and followed by the ‘Concluding Remarks’ section. In the section ‘Methodology, model, and method’ the general agent model is described. There, the random state intervention acts as a catalyst, and the technological level of a leading company represents the fitness of an agent. This is discussed in more detail in the section ‘Inspiration from company market’ along with the pivotal market-based interpretation of our work.

## Manifold multi-spectral critical phenomena: results and discussion

This section presents first- and second-order statistics, which prove the existence of a to date unobserved kind of continuous phase transition characterised by a phenomenon of *multi-spectral* criticality. Characteristic representation of the first-order statistics can be the average population of agents on the lattice. The second order can be the variance of this population—both in the quasi-stationary macrostate of the system.

By the ‘average time-dependent population of agents, we mean the agents’ time-dependent population, $c(t) = N(t)/L^2$, averaged over a statistical ensemble of the macrostate’s replicas obtained by Monte Carlo (MC) simulations, $\langle c(t)\rangle = \langle N(t)\rangle /L^2$, to reduce the statistical fluctuations; it is still a time-independent quantity. Below, we present the method of determining $c_{st}$ (and hence $\langle c_{st}\rangle ~$). This quantity can reach its plateau, $\langle c(t)_{st}\rangle =\langle c_{st}\rangle =const$. The phase transition we consider only for systems whose average number of agents/companies, $\langle N(t)\rangle ~$, has already relaxed to the appropriate plateau $\langle N(t)_{st}\rangle =\langle N_{st}\rangle = const$. In the ‘ Methodology, model, and method’ Section below, we discuss the relaxation of the relative time-dependent average level of technology, $\langle \overline{A(t)}\rangle /F(t)$, showing that it also reaches its plateau, $\left( \langle \overline{A(t)}\rangle /F(t)\right) _{st}=const$, for a long time, where quantity $\langle \overline{A(t)}\rangle ~$ is the average technological level(fitness) of the market(system).

### Quasi-stationary surface

**Figure 1:**
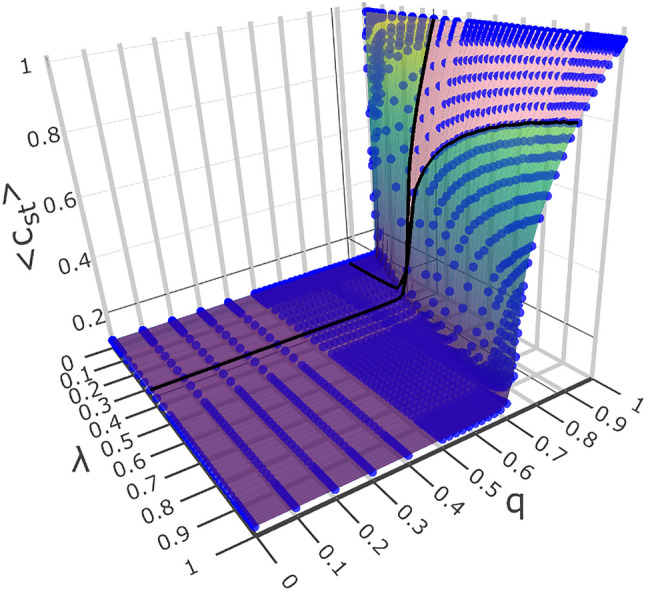
Quasi-stationary 2-dimensional surface (coloured) in variables $(\lambda , q, c_{st})$ obtained from the simulation (at the fixed/typical value of $\eta = 0.5$); quantity $\langle c_{st}\rangle ~$ denotes the mean concentration over the statistical ensemble of MC-simulation replicas. This is the snapshot picture taken from animation. The surface represents the quasi-stationary phase diagram embedded in the three-dimensional space. The bold black curves on the surface are related to the common critical point $(\lambda _c=0.349, q_c=0.879, \langle c_{st}\rangle =0)$, which is somewhat masked by finite-size effects. The black curves result from the intersection of mutually perpendicular planes $(\lambda , q=q_c, \langle c_{st}\rangle )$ and $(\lambda =\lambda _c, q, \langle c_{st}\rangle )$ with the surface—see Fig. 2. The area between these black curves marked in pink—compare projections in Figs. 2–4.

Figure 1 shows a quasi-stationary phase diagram (multicoloured surface) in variables $\lambda , q$, and $\langle c_{st}\rangle ~$ (i.e. agent activity, catalysis level and mean population of agents, respectively), at a fixed value of $\eta$ (i.e. catalysis efficiency).

By the term “quasi-stationary phase diagram,” here we understand the dependence of the plateau of the relaxed average lattice population of agents, $\langle c_ {st}\rangle ~$, on the variables $\lambda$ and *q*. The population reaches a plateau in these variables (with the $\eta ~$ variable value fixed). Below we show that the phase transition scenario plays out in the $\lambda , q$, and $c_{st}$ variables.

### Manifold criticality

**Figure 2:**
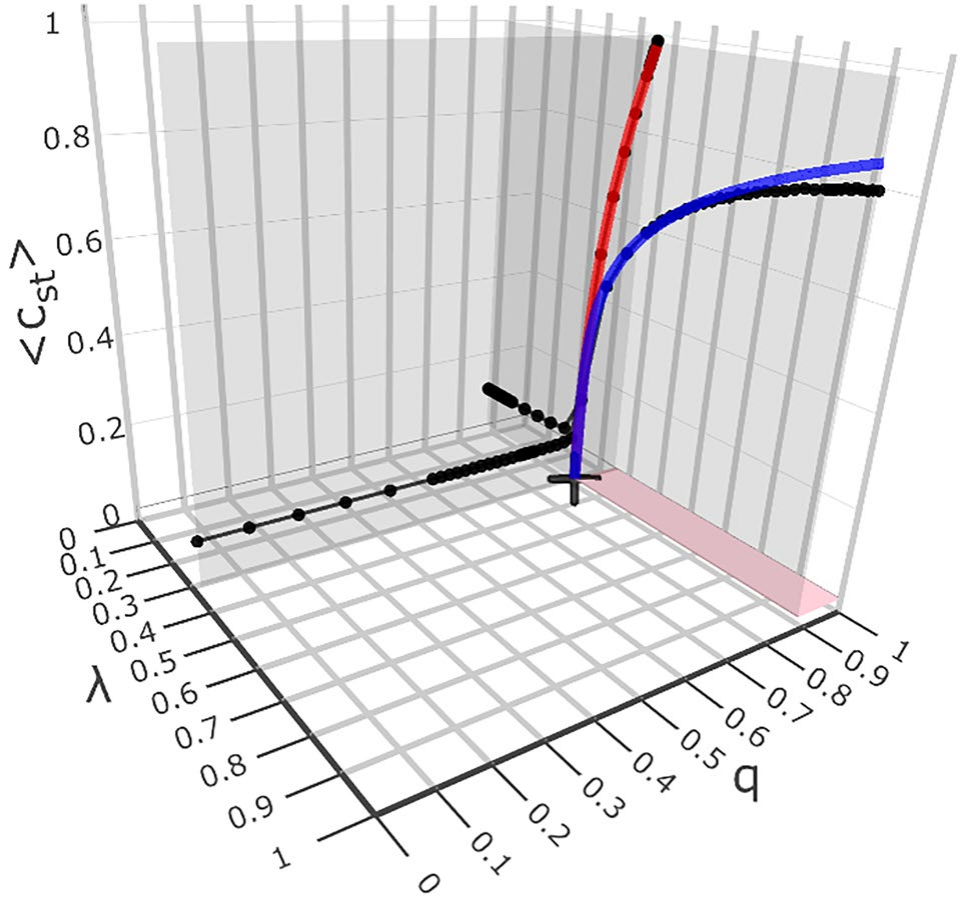
The diagram showing the origin of a critical point marked with sign $`+'$ and located at $(\lambda =\lambda _c=0.349, q=q_c=0.879, \langle c_{st}\rangle =0)$. It is the snapshot from animation. Both black curves lying in mutually perpendicular (grey) planes $(\lambda , q_c, \langle c_{st}\rangle )$ and $(\lambda _c, q, \langle c_{st}\rangle )$ we took from Fig. 1 as the intersection of these planes with the (coloured) surface shown there. The blue and red curves share (to a good approximation) the $`+'$ point. The essential element here is that both curves were suitably fitted to the black curves (in the range just not below this point) using power laws obtained from Eqs. (1) and (2), respectively. Thus, this common point is a multi-spectral critical point. We have shown the course of the power curve on an analogous, exemplary plane lying between the two mentioned above in Fig. 5 below. This curve lies (accurate to the finite-size effect) in the area marked in pink on the surface in Fig. 1. The projection of this area is here (and in Figs. 3 and 4) onto a plane $(\lambda , q, \langle c_{st}\rangle =0)$ marked with the same colour.

**Figure 3:**
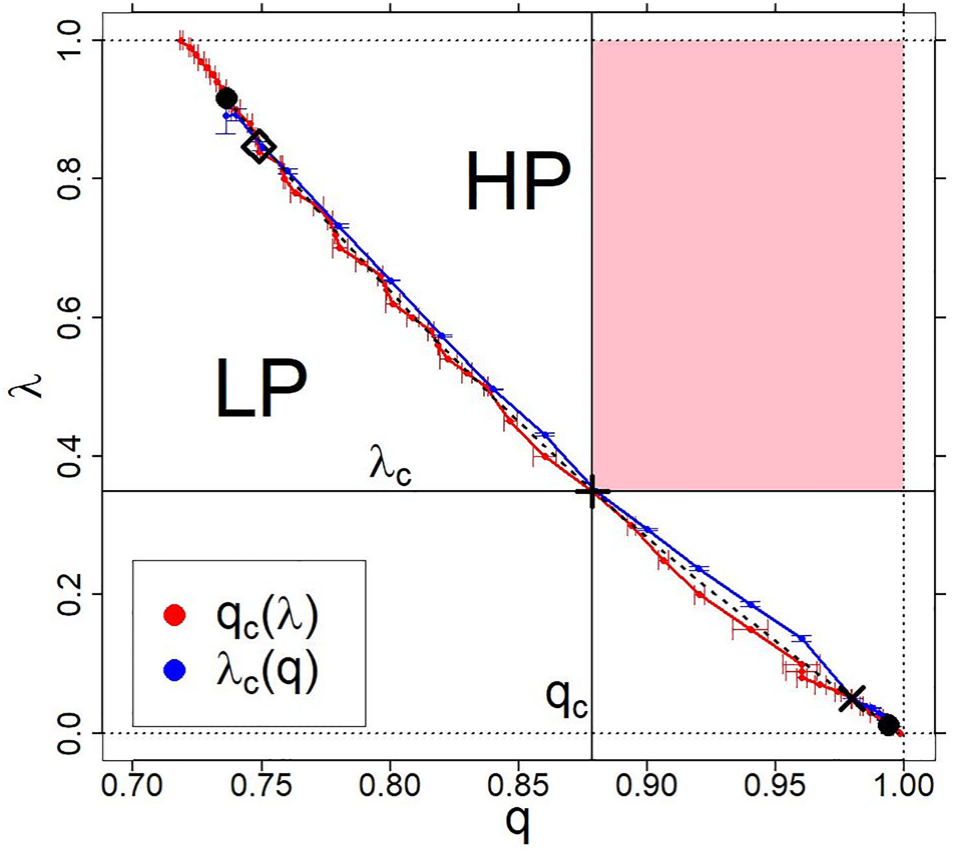
Two close-lying curves on the $(\lambda , q, \left<c_{st}\right>=0)$ plane—blue and red small circles from the simulation with statistical errors marked. The black dashed curve defines the resultant/extrapolated curve between the above-mentioned two. For better visibility, the plot is limited to the $0.69\le q\le 1.0$ range. The common points of both curves constitute the manifold of critical points. Figure 2 shows how to create one of such points, marked by $`+'$. We marked this (example) critical point at the intersection of mutually perpendicular thin black straight lines (horizontal and vertical). These lines form the basis of the grey planes shown in Fig. 2. The rectangular area marked in pink is the area marked with the same colour in Fig. 2. We can move this critical point along the black dashed curve and the structure shown in Fig. 2. Two more example critical points—one identified with a ’$\diamond$’ symbol and one with an ’$\times$’ symbol—are shown for illustration of this. This dashed line is described (to a good approximation) by an equation of the form $\lambda = a-b\; q^{\beta }$, where $a=1.0046$, $b=0.2890$ and $\beta =0.7933$.

To reveal the phase transition, we intersect the surface in Fig. 1 with two mutually perpendicular planes: $(\lambda , q=q_c=0.879,\langle c_{st}\rangle )$ and $(\lambda =\lambda _c=0.349, q,\langle c_{st}\rangle )$, i.e., parallel to the $\lambda$ and *q* axes, respectively. The former plane contains the blue curve, while the latter includes the red one; they are shown in Fig. 2. These coloured curves are the graphical representation of formulas, Eqs. (1) and (2), respectively. We emphasise that the point $(\lambda _c=0.349,q_c=0.879,\langle c_{st}\rangle =0)$, marked with ‘$+$,’ is an arbitrary (typical) point on the critical curve(1-dimensional critical manifold). We show this curve and ‘+’ by top view in Fig. 3. All the other points of this curve were obtained analogously. Thus, these points correspond to the situation after subtracting the ’finite size effect’ because the said power laws cut it off. The important result here is that both curves (blue and red) in Fig. 3 coincide, which allows them to be treated as one curve (with statistical dispersion). We can now systematically analyse the properties of the phase diagram surface shown in Fig. 1.

**Figure 4:**
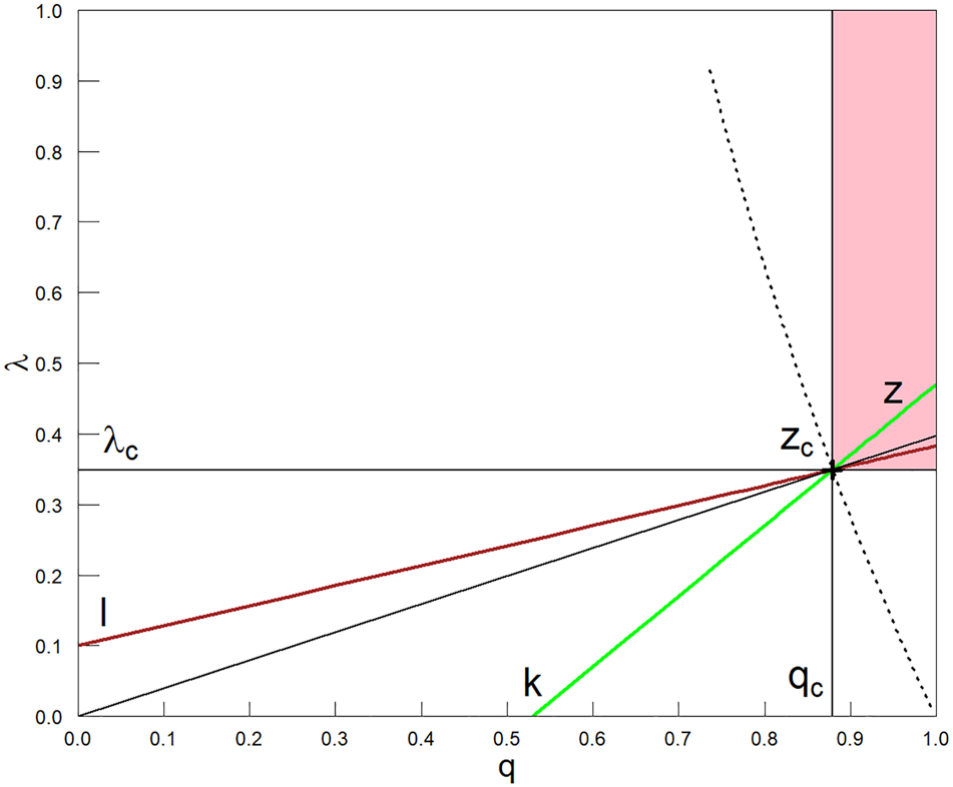
Expanded projection of the phase diagram, represented in the three-dimensional coordinate system $(\lambda , q, \langle c_{st}\rangle )$ in Figs. 1 and 2, to the plane $(\lambda , q, \langle c_{st}\rangle =0)$. It is supported by the projection shown in Fig. 3. The black, thin, mutually perpendicular straight lines intersecting at the point marked by‘+’ are the projection of mutually perpendicular planes $(\lambda _c=0.349, q, c_{st})$ and $(\lambda , q_c=0.879, c_{st})$, which are oriented inwards (to keep the coordinate system clockwise). These planes are grey in Fig. 2. Between the planes we have placed here, for example, three other planes, the projections of which we have marked with diagonal straight lines in green, black, and brown. The parameter $0\le k<q_c$ marks the beginning of the green line, the parameter $0\le l<\lambda _c$ marks the beginning of the brown line, and $k=l=0$ is the beginning of the black demarcation line between the above areas. For example, the green line has been drawn in the figure for $k=0.530$, and the brown line for $l=0.1$. The sign $`+'$ is the intersection of all six curves. Alternatively, the sign is the common intersection of the corresponding five planes projected onto the plane $(\lambda , q, \langle c_{st}\rangle =0)$. The *z* variable specifies the distance of the selected point, for example, on the green line from the beginning of this line. Then, for instance, $z_c=\sqrt{\lambda _c^2+(q_c-k)^2}$.

The diagram in Fig. 2 shows how the critical point, $(\lambda =\lambda _c, q=q_c, \left<c_{st}\right>=0)$, (denoted by ‘$+$’) forms. Both monotonically increasing black curves lying on (grey) mutually perpendicular planes $(\lambda , q=q_c, \left<c_{st}\right>)$ and $(\lambda =\lambda _c, q, \left<c_ {st}\right>)$, were formed as a result of their intersection with the (coloured) phase diagram surface shown in Fig. 1. Below, we show that in our model the mutual location of both planes can be selected so that the above-mentioned black curves have, in principle, a common critical point ‘+.’ However, due to the finite size effect, the location of the ‘+’ point may be too inaccurate. For better localisation (i.e., reducing the finite size effect), we use the following scaling laws: Eqs. (1) and (2). Indeed, points obtained in this way form coincident curves blue and red shown in Figs. 3 and 4. The smooth black dashed curve in Fig. 3 is located between the blue and red curves, thus defining an optimal 1-dimensional critical manifold. Indeed, this curve we also presented in Fig. 4.

The blue curve, fitted in Fig. 2 to the corresponding black dots lying on one $(\lambda , q=q_c, \left<c_{st}\right>)$ of the two grey planes, is captured by the following power/scaling law ^4,32^,

1 $$\begin{aligned} c_{st}(\lambda ; \lambda _c, q_c)= &  \Lambda (\lambda _c, q_c) \nonumber \\&\quad \times&\left( \frac{\lambda -\lambda _c}{1-\lambda _c}\right) ^{\nu (\lambda _c, q_c)}. \end{aligned}$$

The above-given expression describes the critical behaviour (blue curve) solely in variable $\lambda$. It was fitted to part of the visibly rising curve of black dots, giving the critical value $\lambda _c = 0.3491\mp 0.0001$, the critical exponent $\nu =0.219\mp 0.004$, and prefactor $\Lambda =0.868\mp 0.004$. The mutual fit of both curves (black and blue) is suitable for almost the entire range of $\lambda \ge \lambda _c$. A slight deviation occurs near the critical threshold due to the finite-size effects. Also, deviations occur for large values of $\lambda$, which is irrelevant from the point of view of a critical phenomenon.

The complementary power law,

2 $$\begin{aligned} c_{st}(q; \lambda _c, q_c)= &  Q(\lambda _c, q_c) \nonumber \\&\quad \times&\left( \frac{q-q_c}{1-q_c}\right) ^{\beta (\lambda _c, q_c)}, \end{aligned}$$

describes the critical behaviour in the $q\ge q_c$ variable (same as above for $\lambda$ variable). We fitted the course of the red curve in the $(\lambda =\lambda _c, q,\langle c_{st}\rangle )$ plane to the corresponding black dots. The values of fitted quantities are as follows:  $q_c = 0.8787\mp 0.0002$, prefactor $Q=0.979\mp 0.004$, and critical exponent $\beta =0.345\mp 0.008$, where we took the $\lambda _c=0.3491\mp 0.0001$ value obtained above.

The blue and red curves share the $`+'$ point thanks to the appropriate mutual location of both perpendicular planes. The essence here is that both curves were suitably fitted to the corresponding black curves using power laws given by Eqs. (1) and (2), respectively. Thus, this common point is critical of the multi-spectral type; additional arguments are presented below. Moreover, the critical exponents $\nu ~$ and $\beta ~$ characterising this critical point, controlling the boundary power laws (1) and (2), respectively, have offsets from the spectrum of critical exponents described later in the main text and shown in summary Fig. 10. For now, the cause of both offsets is an open question which applies to the entire 1-dimensional critical manifold.

In Fig. 3, we present two close-lying(coincident) critical manifolds (blue and red) created correspondingly by blue and red curves described in Fig. 2. That is, scaling regions of both critical manifolds overlap the entire length of the blue (shorter) curve, extending from point $\lambda _c (q = 0.736) = 0.892$ to point $\lambda _c (q = 0.995) = 0.010$. Let us add that the red curve extends from point $q_c(\lambda = 1) = 0.719$ to point $q_c(\lambda = 0.001) = 0.998$ (errors in determining these quantities are up to the third significant digit after the decimal point). The space between both critical curves defines a 1-dimensional manifold of critical points (the black dashed curve). The slight split of blue and red curves results mainly from statistical errors and finite-size effects.

### Multi-spectral critical behaviour of $c_{st}$

We can now extend the data scaling with Eqs. (1) and (2) concerning boundary planes $(\lambda , q=q_c, c_{st})$ and $(\lambda =\lambda _c, q, c_{st})$, respectively, to any oriented plane $(\lambda , q, c_{st})$ between them perpendicular to $(\lambda , q, c_{st}=0)$ plane (see Fig. 4 for illustration). The basis of the $(\lambda (q), q,c_{st})$ plane, e.g., the green straight line placed on $(\lambda , q,c_{st}=0)$ plane, we can present in variables $(\lambda ,q)$ in the form of the linear relation between them,

3 $$\begin{aligned} \lambda (q)= \lambda _c~\frac{q-k}{q_c-k}\ge 0, \end{aligned}$$

where $0\le k<q_c$ and $k\le q\le 1$. For convenience, we only use non-negative values for the *k* directional parameter—the consequences of which we discuss below. Equation (3) is obtained assuming that the green straight line should pass through two points: $(\lambda = 0, q = k)$ and $(\lambda _c, q_c)$—we marked this latter point in Fig. 4 by sign $`+'$. It is the same critical point we marked in Figs. 2 and 3. It should be emphasised that the point marked by $`+'$ is only an example of a critical point on the dashed critical curve in Fig. 3. We might choose any other point on this curve for our consideration such as the ones denoted with $\diamond$ and $\times$.

**Figure 5:**
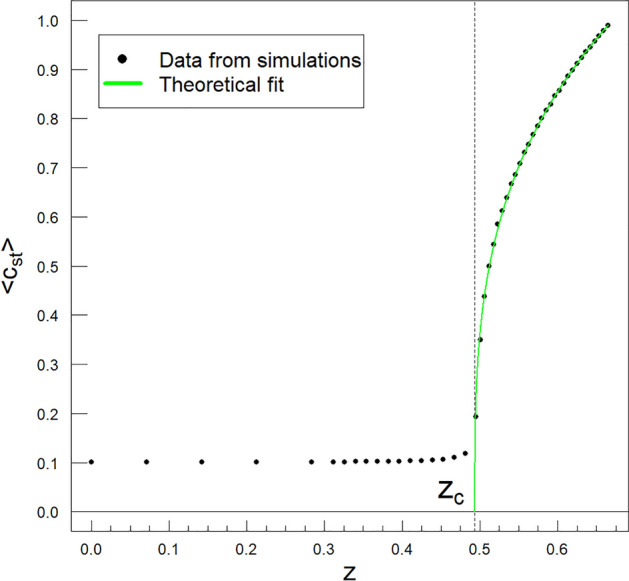
The dependence of the order parameter $c_{st}$ vs. *z*. The solid curve (in green) we obtained from the formula Eq. (8) by fitting it to data (black dots) from computer simulations. These data were additionally averaged over the statistical ensemble of replicas of the initial simulation (numerical experiment). Therefore, we denote them by $\langle c_{st}\rangle ~$; hence, the plot’s vertical axis has such a notation. From this fit we obtained $\Theta = 0.990 \mp 0.001$ and $\zeta = 0.309 \mp 0.002$ with $k = 0.530$ and $z_c = 0.494$, i.e., $q_c = 0.879$ and $\lambda _c = 0.349$. These parameters locate the multi-spectral critical point as shown (by the $'+'$ sign) in Figs. 2–4. The green curve is drawn along the green straight line in Fig. 4, starting from $`+ '$. Fits for points marked with $\diamond$ and $\times$ in Fig 3 look analogical and are not shown.

The basis of the other $(\lambda , q(\lambda ), c_ {st})~$ plane is the brown curve in Fig. 4. Its equation in the variables $(\lambda , q)~$ takes the following linear form,

4 $$\begin{aligned} q(\lambda ) = q_c~\frac{\lambda -l}{\lambda _c-l}\ge 0, \end{aligned}$$

where $0\le l<\lambda _c$ and $l\le \lambda \le \lambda _f=l+\frac{\lambda _c-l}{q_c}$, having $\lambda _f=\lambda (q=1)$. For convenience, we have limited the *l* directional parameter range to non-negative values, similar to the *k* parameter. This type of double parameterisation is the price we pay for using non-negative parameters *k* and *l*. We obtained Eq. (4), assuming that the straight brown line should pass through two points: $(\lambda =l, q=0)$ and  $(\lambda _c, q_c)$. The diagonal black straight line meeting Eqs. (3) and (4) for $k=l=0$ is the boundary line separating both methods of parameterisation discussed above. Eqs. (3) and (4) are intended to offer useful parameterisation for straight lines passing through $(\lambda _c, q_c)~$ to define (below) the compact variable *z* conveniently. The plane $(\lambda (q), q,c_{st})$ based, for example, on the green straight line shown in Fig. 4 intersects the (multicoloured) surface presented in Fig. 1, giving the black dots shown in Fig. 5.

### Multi-spectral critical relaxations

**Figure 6:**
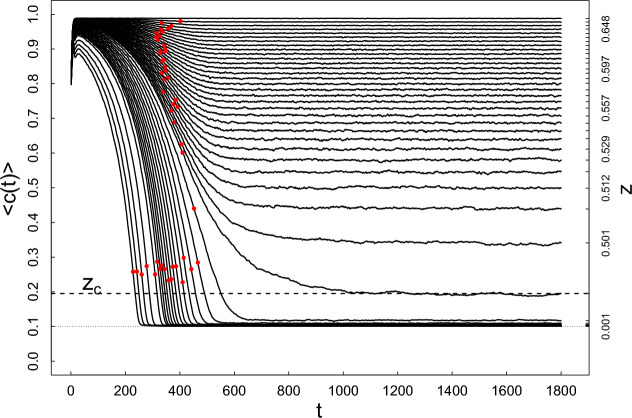
The time-dependence of the population of companies on the lattice, $\left<c(t)\right>=\left<N(t)\right>/L^2$ (averaged over statistical ensemble—left linear scale) and the corresponding *z* (right non-linear scale). Time *t* is measured here in MCS/site units and lattice size $L=20$. The dotted horizontal line corresponds to $c_{min}=N_{min}/L^2$, while the dashed horizontal line concerns the multicritical point $z=z_c$ that is, it refers to the scale on the right. The curves were determined for $z<z_c$ and $z\ge z_c$. Plateaus are reached slower or faster by the various visible curves. The red points on these curves indicate their inflection points. All simulations were performed for fixed $\eta =0.5$.

In Fig. 6 we present the time-dependence of the population of agents on the lattice, $c(t)=N(t)/L^2$ obtained by MC simulations (for details see ‘Methodology, model, and method’ Section below and  Supplementary Information. The curves $\left\langle c(t)\right\rangle$ obtained were determined for the points lying on the green diagonal straight line shown in Fig. 4. They result from averaging the corresponding curves over a statistical ensemble of 400 replicas i.e., single independent repetitions of a given system simulation. As shown in Fig. 6, the plateaus are reached at different rates. Plateaus for small and large companies’(agents’) populations on the lattice have the highest rates. On the other hand, plateaus for intermediate populations (i.e., in the vicinity of $z_c$) are reached slower. The relaxation time (as the reciprocal of the rate) has a local maximum in the variable *z*. Here we only discuss the properties of the system after it has reached a plateau for different values of the plateau. A striking feature of the relaxation spectrum presented in Fig. 6 is the apparent dependence of its plateaus on *z*, that is, both on the level of state intervention(catalyst), *q*, and on the level of company(agent) activity, $\lambda$. The main goal of this work is investigating this relationship.

**Figure 7:**
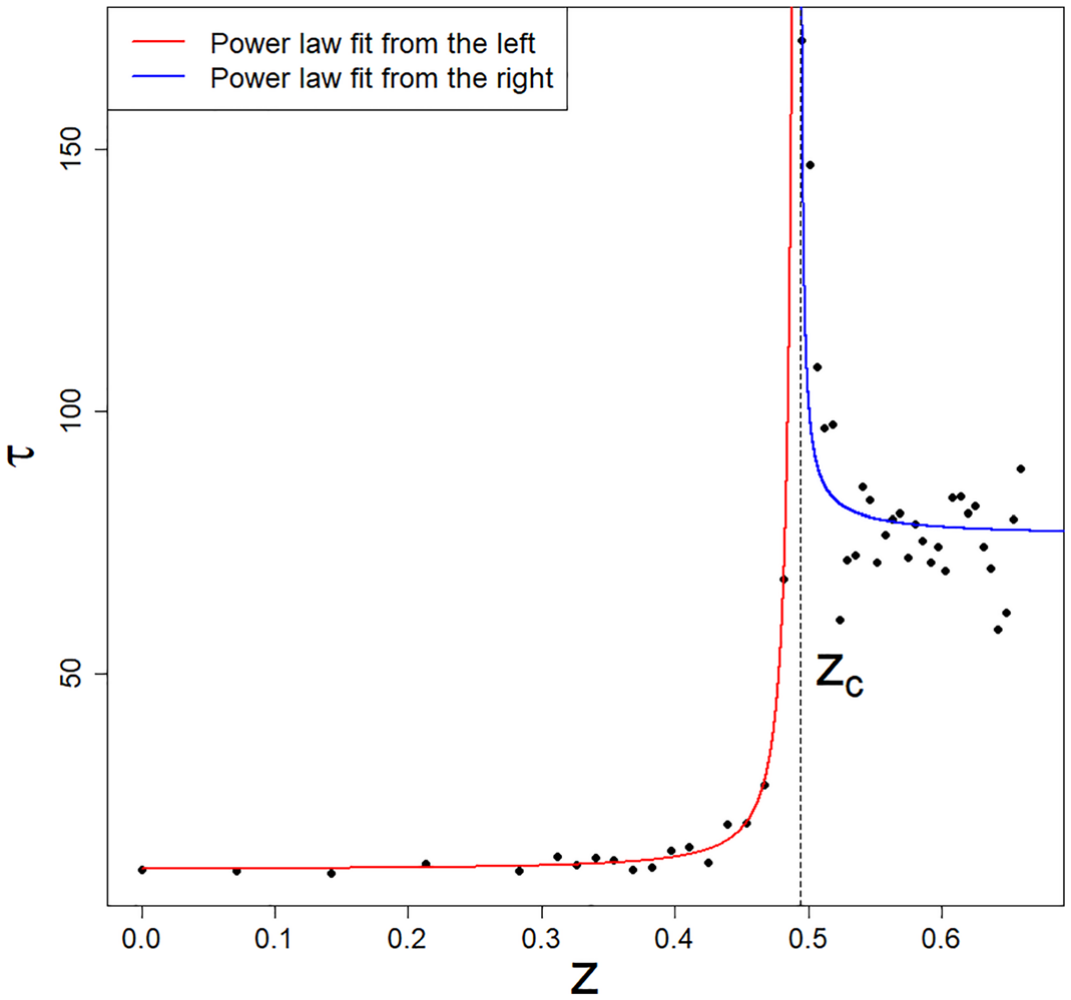
The dependence of the integrated non-linear relaxation time, $\tau$, vs *z* at fixed value of $\eta =0.5$. A visible divergence of $\tau$ at the critical threshold $z_c=0.4937516$ defines our model’s multicritical nonlinear growth and slowing down effects in the *z* variable. The black dots result from the MC simulation, while the formula Eq. (7) gives the solid lines. We can talk about the multicritical feature of this effect because we have the divergence of $\tau$ for both (red and blue curves) critical 1-manifolds presented in Fig. 3. This is what justifies the name “multicritical dynamics/kinetics/relaxation” we observe. Fits for points marked with $\diamond$ and $\times$ in Fig. 3 look analogical and are not shown.

In Fig. 7, the integrated non-linear relaxation time dependence on *z* variable is shown at fixed $\eta$. The relaxation time we can define as follows,

5 $$\begin{aligned} \tau = \int _{t_0}^{\infty }\phi (t)dt, \end{aligned}$$

where $t_0\ge t_{ip}$, while $t_{ip}$ locates in time an inflection point (see red dots on curves in Fig. 6), while the non-linear relaxation function $\phi (t)$ takes the form,

6 $$\begin{aligned} \phi (t)=\frac{\langle c(t)\rangle -\langle c_{st}\rangle }{\langle c(t_0)\rangle -\langle c_{st}\rangle }, \end{aligned}$$

where brackets $\langle \ldots \rangle$ mean the averaging over a time-dependent statistical ensemble of replicas. Thus, we have defined the global relaxation mode for the company market (where Eq. (24)) defines an example of the local mode).

### Spectral multicritical growth and slowing down

The result shown in Fig. 7 is the pillar of this work and shows that there is $z = z_c$ for which the integrated non-linear relaxation time, $\tau$, diverges in the *z* variable. Thus, it reveals this variable’s critical growth and slowing down effects. This is supported by the observation that this divergence is of the power-law nature. Namely, solid lines on the plot are described by the following (dichotomous) formula,

7 $$\begin{aligned} \tau _{\mp }(q)=A_{\mp }\left| 1-\frac{z}{z_c}\right| ^{-\xi _{\mp }}+B_{\mp }, \end{aligned}$$

where the sign $`-'$ concerns the left edge of the peak (i.e., the situation for $z<z_c$ described by the red curve) and the sign $`+'$ concerns the right edge (i.e., the situation when $z>z_c$ described by the blue curve), exponents $\xi _{\mp }(>0)$ are the critical dynamic ones, $A_{\mp }$ denote prefactors/amplitudes, and $B_{\mp }$ denote the background level. In Table 1 we have compiled the obtained values of quantities (exponents, prefactors, and background coefficients). Figure 6 presents the critical threshold we took from fit.

**Table 1:** Adjusted parameter values.

Quantity	Index −	Index +
$\xi _{\mp }$	$1.52452\mp 0.09661$	$0.9167\mp 0.3115$
$A_{\mp }$	$0.19902\mp 0.07111$	$0.13\mp 0.22$
$B_{\mp }$	$12.65268\mp 0.55088$	$76.0835\mp 4.7216$

**Figure 8:**
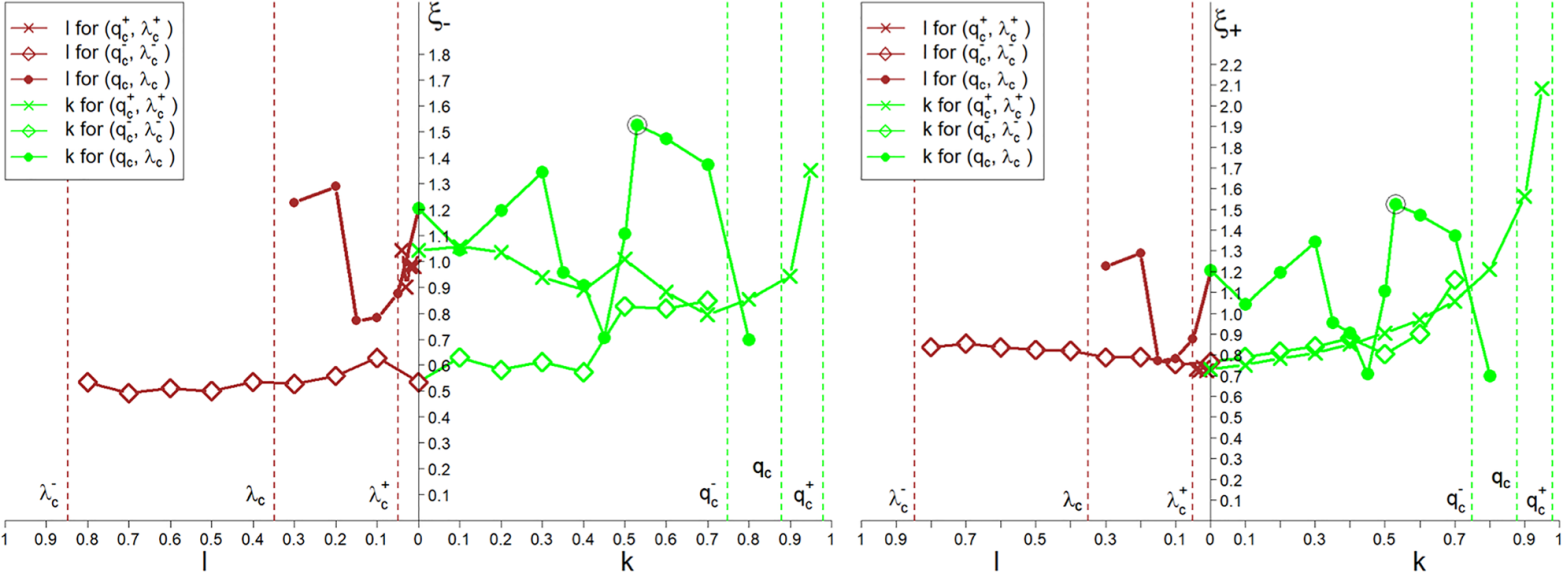
Spectra of both multicritical exponents $\xi _{\mp }$ (appearing in Eq. (7)). The *k* and *l* control parameters are in the ranges $0\le k\le q_c$ and $0\le l\le \lambda _c$, respectively. There is a large variability of the exponent $\xi _-$ compared to the exponent $\xi _+$. Spectra are shown for all the three critical points marked with $\diamond$, $+$ and $\times$ in Fig. 3 and the *z* range on both sides of the criticality threshold $z_c$—the left plot on the left (i.e., for $z< z_c$) and the right one on the right side (i.e., for $z\ge z_c$).

The plots in Fig. 8 show the spectra of both critical exponents $\xi _{\mp }$. The existence of these spectra confirms the spectral multicritical nature of the continuous phase transformations in the system.

### Phase classification

The dependence of the integrated non-linear relaxation time $\tau$ (see Fig. 7) enables us quantitatively to differentiate populations of agents/companies on the lattice. These correspond to distinct areas in the quasi-stationary phase diagram shown in Fig. 1 and in its projection shown in Fig. 3. These areas can be intuitively described as follows:

Low The low population/concentration area (LP, defined by the almost horizontal part of the phase diagram surface in Fig. 1), where the probability of meeting two agents is residual—agents behave there as independent of each other, e.g., like free particles in dilute lattice gas. Although the agents almost do not block each other’s displacements between lattice sites, the intensity of their mutual interactions is low. This means that their effective activity roughly expressed by $\lambda c_{st}$ is also tiny. We refer to this area of the phase diagram low-concentric or low-effective activity, whereby the term ’effective activity’ means the intensity/frequency of interactions between agents leading to mergers of companies or the creation of new companies (in the form of spin-offs).
High The high population region (abbreviated by HP) is separated from the LP by a narrow transition region due to finite size effects—this region gets narrower the greater the lattice size. In this area, there is a rapid increase in the population of agents, resulting in a significant increase in their effective activity. This area can be divided into two parts:
  1. An area of multiscaling, i.e., multi-spectral critical region, where the power-law increase of agents lattice population is in force—there is a multi-channel mutual interaction of agents, including an autocatalytic interaction/dependence. This area is the scaling region.
  2. An area of high population where the probability of the agents meeting is high, i.e., outside the scaling area. There, the number of vacancies on the lattice is relatively small.

We use the above classification in the subsections below, i.e., the behaviour of the order parameter (or agent population on the lattice, see Fig. 1). Since we are dealing with a continuous phase transition, we discuss singularities of variance at multi-spectral critical points. We confirm the presence of singularities in this phase transition, i.e., transition between the inactive and active phase.

### Divergence of variance

**Figure 9:**
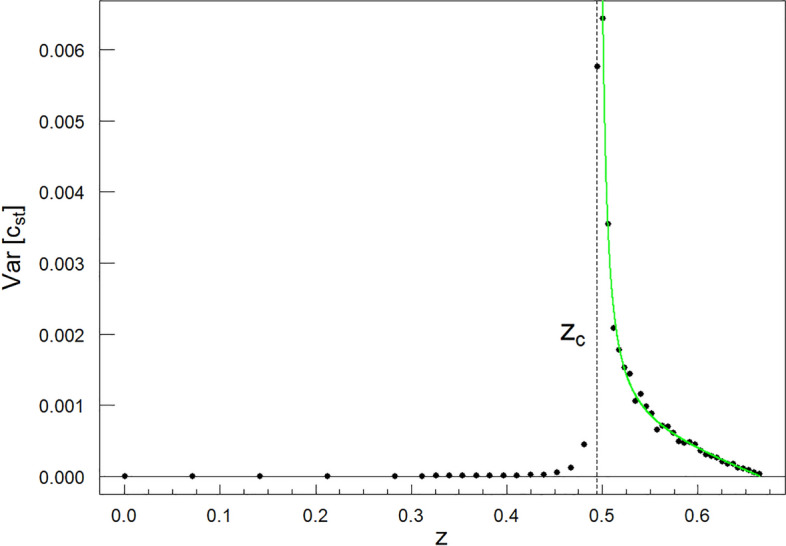
The diverging variance $Var[c_{st}(z; k, \lambda _c, q_c)]$ vs. *z*. The singularity of variance at the critical point $z_c = 0.494$, as marked in Fig. 5, is well seen. Parameters characterising the singular component in Eq. (16) are as follows: $\mu =1.286 \pm 0.065$ and $V=0.00010\mp 0.00002$. While the parameters characterising the linear background in the mentioned formula are as follows: $a=0.0050\mp 0.0007$ and $b=0.0032\mp 0.0004$. This result and the one presented in Fig. 5 indicate that we are dealing with a continuous phase transition at $z_c$. Fits for points marked with $\diamond$ and $\times$ in Fig. 3 look analogical and are not shown.

The green curve in Fig. 5 represents the fit to the black dots of the $c_{st}$ vs. $z\ge z_c$ given by one-dimensional scaling (power law),

8 $$\begin{aligned} c_{st}(z;k,\lambda _c, q_c)= &  \Theta (k,\lambda _c, q_c) \nonumber \\\times &  \left( \frac{z-z_c}{z_f-z_c}\right) ^{\zeta (k, \lambda _c, q_c)},~z\ge z_c, \end{aligned}$$

where the compact scaling variable, *z*, is given for $0\le k<q_c$ by the following formula,

9 $$\begin{aligned} z= &  \sqrt{(q-k)^2+\lambda ^2}=\sqrt{(q-k)^2+~\lambda _c^2\frac{(q-k)^2}{(q_c-k)^2}} \nonumber \\= &  (q-k)\sqrt{1+\left( \frac{\lambda _c}{q_c-k}\right) ^2}, \end{aligned}$$

with its critical value $z_c=\sqrt{(q_c-k)^2+\lambda _c^2}$, and

10 $$\begin{aligned} z_f=z(q=1)=(1-k)\sqrt{1+\left( \frac{\lambda _c}{q_c-k}\right) ^2}, \end{aligned}$$

obtained from Eq. (9). Thus, the compact variable *z* measures the distance of a given point located on the green straight line from its beginning (the point $(\lambda =0, q=k, \left<c_{st}\right>=0)$) along this line. To simplify the notation, we do not introduce additional indexing of the *z* variable and the $z_c$ and $z_f$ parameters using the directional parameter *k* and *l*.

For the complementary case of $0\le l<\lambda _c$ we obtain

11 $$\begin{aligned} z= &  \sqrt{q^2+(\lambda -l)^2}=\sqrt{(\lambda -l)^2 + q_c^2\frac{\left( \lambda -l\right) ^2}{\left( \lambda _c-l\right) ^2}} \nonumber \\= &  (\lambda -l)\sqrt{1+\left( \frac{q_c}{\lambda _c-l}\right) ^2}, \end{aligned}$$

with its critical value  $z_c=\sqrt{(\lambda _c-l)^2+q_c^2}$. As before, the *z* variable measures the distance of a given point placed on the straight brown line defined by the direction parameter *l* from the beginning of this line (i.e., from the point $(\lambda =l, q=0, \left<c_{st} \right>=0 )$) along its direction.

In this case, we can formulate a one-dimensional power law complementary to Eq. (8),

12 $$\begin{aligned} c_{st}(z;l,q_c,\lambda _c)= &  \Theta (l,q_c,\lambda _c) \nonumber \\\times &  \left( \frac{z-z_c}{z_f-z_c}\right) ^{\zeta (l,q_c,\lambda _c)},~z\ge z_c, \end{aligned}$$

where both the prefactor $\Theta ~$ and the multi-spectral critical exponent $\zeta ~$ depend explicitly on *l* instead of *k*. Next, (from Eqs. (11) and (4)) we obtain,

13 $$\begin{aligned} z_f=z(q=1)=\sqrt{1+\left( \frac{\lambda _c-l}{q_c}\right) ^2}. \end{aligned}$$

The following continuity boundary condition applies,

14 $$\begin{aligned} \Theta (k=0, \lambda _c, q_c)=\Theta (l=0,\lambda _c,q_c), \nonumber \\ \zeta (k=0,\lambda _c, q_c)=\zeta (l=0, \lambda _c, q_c). \end{aligned}$$

**Figure 10:**
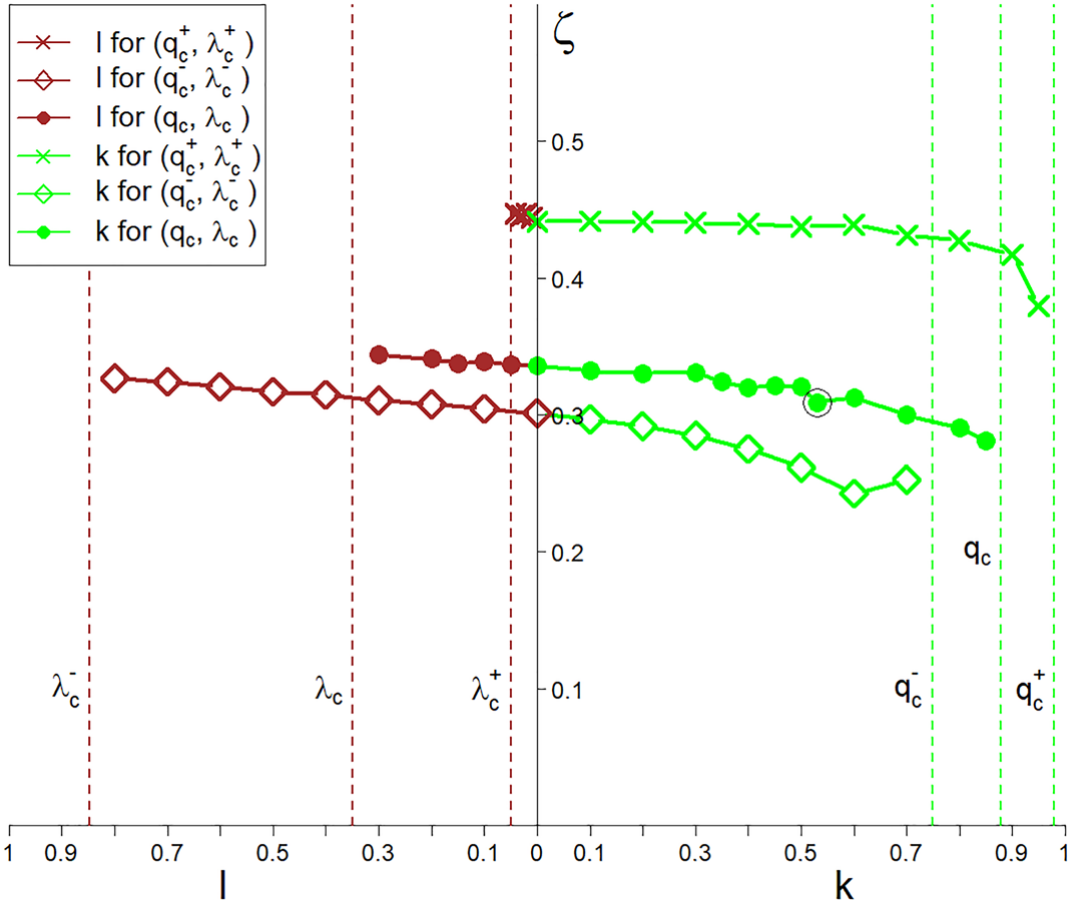
The plots of the spectra of the $\zeta ~$ exponent (appearing in Eqs. (8) and (12)), which depend on the parameters *k* and *l*, respectively. Spectra are shown for all the three critical points marked with $\diamond$, $+$ and $\times$ in Fig. 3.

We limit ourselves to two opposite areas of the $(\lambda ,q)$ variables, namely to $(0\le q<q_c)$ $\times ~$ $(0\le \lambda <\lambda _c)$ and  $(q_c\le q\le 1.0)$ $\times ~$ $(\lambda _c\le \lambda \le 1.0)$. It is worth emphasising that the proposed multiscaling laws, Eqs. (8) and (12), describe universality classes indexed with the *k* and *l* parameters. The plot in Fig. 10 shows the non-uniform sub-spectrum of the $\zeta ~$ exponent. The borderline scaling laws described by Eqs. (1) and (2) are complementary to Eqs. (8) and (12) .

**Figure 11:**
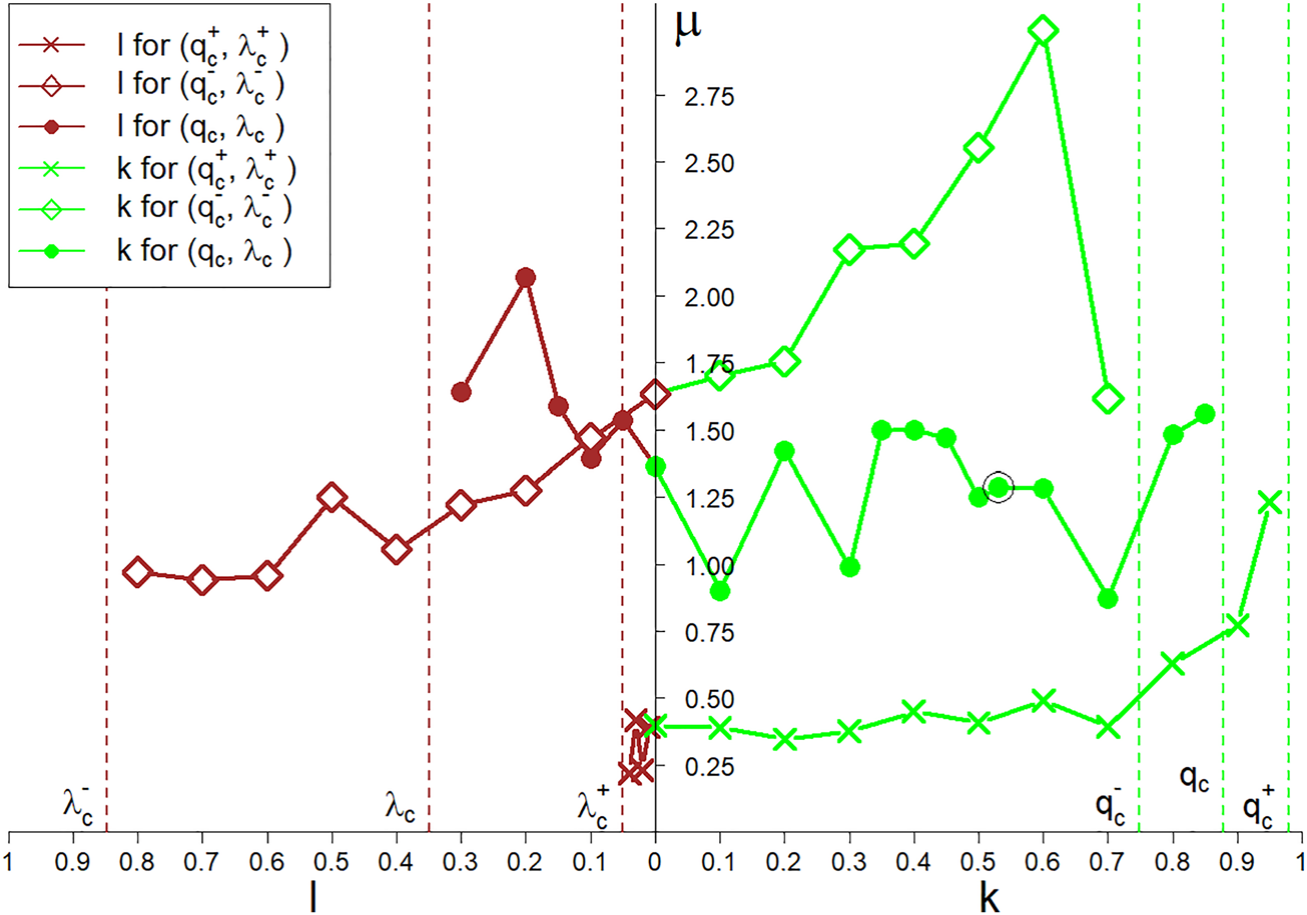
Spectra of the $\mu ~$ exponent (appearing in Eq. (16)), which show significant oscillations. This is related to the multi-spectral critical point $(\lambda _c, q_c, \left<c_{st}\right>=0)$ marked by $`+'$ in Figs. 2–4. Spectra are shown for all the three critical points marked with $\diamond$, $+$ and $\times$ in Fig. 3.

To summarise this subsection, we note that in our case, the order of the phase transition remains unchanged—we are dealing with a continuous phase transition. The essence of our phase transition, different from multicriticality, is shown in Fig. 4. The dashed line is the critical manifold, the same as in Fig. 3.

### Divergence of susceptibilities

The scaling, Eqs. (1) & (2), and multiscaling Eqs. (8) & (12), laws discussed above lead to divergence of susceptibility at the criticality threshold $(\lambda _c, q_c, \left<c_{st}\right>=0)$. We define susceptibility, $\chi ~$, as a partial derivative,

15 $$\begin{aligned} \chi = \frac{\partial c_ {st}}{\partial y}, \end{aligned}$$

where $y = \lambda$ and *q*, respectively, for expressions Eqs. (1) and (2), and $y=z$ for expressions Eqs. (8) and (12). This definition results in susceptibility divergence because the values of the critical exponents $\nu (\lambda _c,q_c)$, $\beta (\lambda _c,q_c)$, $\zeta (k,\lambda _c, q_c)$, and  $\zeta (l, \lambda _c, q_c)$ are less than 1 in the full range of $\lambda _c$, $q_c$, *k*, and *l*. Exemplary (typical) values of the $\nu ~$ and $\beta ~$ exponents are given in the subsection above, and the $\zeta ~$ exponents in Fig. 10 above (while the single value of $\zeta ~$ is given in the caption under Fig. 5). The course of susceptibility $\chi ~$ vs. *z* is similar to the course of variance in Fig. 9. The critical exponent controlling the descent of the right slope of the susceptibility peak has the form of exponent $1-\zeta >0$. The figure also applies to each point of the critical manifold.

### Diverging fluctuations

In the above subsections, we indicated the conditions necessary for continuous phase transitions to appear. This was the behaviour of the population—or the order parameter. We also noticed the divergence of the susceptibilities directly related to the above mentioned behaviour. In this subsection, we indicate the singular behaviour of the variance of the population, $Var[c_{st}]$, (see Fig. 9 for details) as a sufficient condition for the existence of a continuous phase transition and multi-spectral critical phenomena. A variance sub-spectrum consisting of critical exponents co-creates the multi-spectra, which characterises the multi-spectral criticality (see Fig. 11 for details).

The multi-scaling hypothesis indexed, for example, by the *k* parameter takes the following form for $z\ge z_c$:

16 $$\begin{aligned} &  Var[c_{st}(z; k, \lambda _c, q_c)] \nonumber \\= &  \langle \left[ c_{st}(z; k, \lambda _c, q_c)\right] ^2\rangle - \langle c_{st}(z; k, \lambda _c, q_c)\rangle ^2 \nonumber \\= &  V(k, \lambda _c, q_c)\cdot \left( \frac{z-z_c}{z_f-z_c}\right) ^{-\mu (k, \lambda _c, q_c)} \nonumber \\+ &  L(z; k, \lambda _c, q_c), \end{aligned}$$

where $z_c=z_c(k, \lambda _c, q_c)$ and $z_f=z_f(k, \lambda _c, q_c)$ appeared in the description of Eq. (9) and in Eq. (10), respectively. Moreover, the background linear function $L(z; k, \lambda _c, q_c)=-a(k, \lambda _c, q_c)\cdot z+b(k, \lambda _c, q_c)$, where coefficient $a>0$. To get the analogous scaling hypothesis indexed by the *l* parameter, it is formally enough to replace the *k* parameter with the *l* parameter everywhere in the above equation.

In Fig. 9, we present the diverging dependence of the variance $Var[c_{st}(z; k,\lambda _c, q_c)]$ vs. *z* for the multi-spectral critical point, which in Figs. 2–4 has been tagged with $`+'$. Each critical point $(\lambda _c, q_c, \left<c_{st}\right>=0$) belonging to the critical manifold is associated with a family of vertical planes. These contain the power laws described by equations Eqs. (1) & (2) and (8) & (12). Hence, each family of these planes is associated with a given level/order of the statistic to form a spectrum of critical exponents. One can move from one plane to another by continuously changing the values of the *k* or *l* parameters. Therefore, each critical point along the critical manifold possesses a spectrum of critical exponents and for this reason the critical manifold becomes multi-spectral.

## Concluding remarks

To the best of our knowledge, this type of rich scaling phenomena along a nonlinear manifold has to date not been observed in the science of complexity, including agent-based modelling, entailing econophysics and sociophysics. More specifically, we built a numerical, heterogeneous agent-based model inspired by the market of competing companies in the presence of random state interventionism. This interventionism serves as a catalyst operating in an egalitarian manner, i.e., regardless of the technological level(fitness) of companies and market share(rank). We examined the behaviour of heterogeneous agents in the semi-stationary macrostates of the system. We observed in these macrostates a new type of non-equilibrium continuous phase transitions, generating the set of critical thresholds in the form of a multi-spectral 1-dimensional critical manifold. This manifold’s essential feature is that every critical point is associated with at least four different types of sub-spectra of critical exponents responsible for (i) the power-law behaviour of the order parameter, (ii) diverging susceptibility, (iii) variance divergence, and (iv) growth & slowing-down effects.

Every critical exponent characterises a different curve in the macrostates’ space on which the system can reach a given multi-spectral critical point. For example, it can be characteristic of the growth & slowing-down effects concerning the system’s relaxation to semi-stationary macrostates in the scaling area. As the system approaches the critical threshold, the observed divergence of the relaxation time within the scaling region is one characteristic and the telling system behaviour for a continuous phase transition.

We chose to study an egalitarian variant of our model, i.e., the one in which no agent is preferred initially (we treat this as the rule of no preference). As it transpired, this variant has the broadest spectrum of populations’ plateaus and, what is the most significant, it leads to the multi-spectral critical phenomena depending on the compact variable *z* (containing the exogenous random level of catalyst (intervention), *q*, and endogenous single agent’s(company’s) conditional activity level $\lambda$). The fitness of the agent (e.g., technological level of the company) controls the local dynamics/kinetics of the agent in our model^33–36^—the agent/company tries to achieve the leader’s fitness (technological level), competing for survival. We treat this fitness of the leader as a deterministic, time-dependent exogenous field acting on the system.

From the point of view of the thermodynamics of the lattice gas or Ising magnetic system, the variable $c_{st}$ can play the role of the order parameter—concentration/population of lattice gas particles or magnetisation. There is well-known relation between Ising spin, *s*, and the (lattice gas) particle occupation number, *c* (equals 0 or 1), in the form $s=c-1/2$., $\lambda$ could be an analogon of the thermodynamic activity, *q* the analogon of the hydrostatic pressure applied to the system, albeit this analogy is incomplete because *q* is random. In contrast, the hydrostatic pressure is not. The analogy which fully holds here is that both quantities are exogenous and $\eta ~$ is the analogon of a permeability. We have shown that the phase transition scenario plays out in the $\lambda , q$, and $c_{st}$ variables (at a fixed $\eta$).

We would like briefly to address the differences and similarities of our situation relative to those containing multicriticality. At the outset, the critical difference stems from multi-spectral phenomena occurring in quasi-stationary macrostates. At the same time, multicriticality occurs in macrostates of statistical equilibrium of systems. However, in our case, we are dealing only with a single 1-dimensional critical manifold. In physics, even when dealing with multicriticality, it either appears for the boundary points of a single critical manifold or as a result of connecting or crossing various critical manifolds. These points have different properties from those identified in our case.

We focused on systems with curves of critical points and not isolated critical points. Several critical phenomena relate to the *n*-dimensional critical manifolds, where $n\ge 1$. The critical curves sometimes intersect with other transition curves or branches of different phases or phase borders, generating multicritical intersection points. Multicritical points can also locate/terminate on the $(n-1)$-dimensional border of the *n*-dimensional critical manifold. However, systems are still critical at this border. Multicritical points are unique points in thermodynamic or other systems’ parameter/variable space with a continuous phase transition. A single critical exponent no longer describes the critical behaviour at a multicritical point. Moreover, the critical exponents can change abruptly at these points as the control parameter/variable is varied. Hence, the points on the borders usually belong to a different class of universality than those realised within a bulk part of the critical manifold.

Furthermore, in our situation, the semi-critical curves (intersecting the 1-dimensional critical manifold) lie entirely in one phase on the phase diagram. Each semi-critical curve has a power law associated with it, and many such curves end at the same critical point. This means many critical exponents (i.e., a continuous local spectrum of critical exponents) characterise a given critical point—we call it a multi-spectral critical point. Indeed, the current definition of the multicritical point does not cover our situation. However, the multicriticality known so far bears similarities to our situation because multicritical and multi-spectral critical points are characterised by many critical exponents, and not by a single one, as in the case of the usual critical point.

Criticality as a generic phenomenon is a signature of complex systems that are the scene of the struggle of opposites. Balancing these opposites is necessary (at least) for a quasi-stationary macrostate to define. However, more is needed to obtain the critical curve or—in general—the *n*-dimensional critical manifold. For example, for a market of competing companies involving random state interventionism, we deal (in the scaling region) with a single balance condition generating the law of companies’ conservation. This law describes balancing the companies’ number emerging in the market with the number of companies disappearing. In our model, two stochastic processes (expressed in terms of probability currents) are responsible for the disappearance of companies from the market: (i) a merger of companies, as a result of which two companies become one, and (ii) bankruptcy of companies, i.e., the disappearance of companies from the market. In the quasi-stationary macrostate, both these destructive processes are balanced by the company creation process. Two companies can create (with a given probability) a spin-off, i.e., a new company related to them.

However, on the curves of critical points (which we mainly consider), the single balance condition mentioned above breaks down into two special balance conditions corresponding to different balancing paths: (i)The first path corresponds to (a) balancing the central part of the probability current of company creation with (b) the central part of the probability current of its annihilation (associated with the merger of companies and the full probability current of bankruptcy of companies). (ii)The second path is related to balancing the residual parts of the currents of the probabilities of creating and annihilating companies (already without the participation of bankruptcies). The transition from the single balance condition to two specific balance conditions can be seen as a symmetry-breaking phenomenon characterising critical phenomena considered in this work.

Our approach is a modern variant of the game of life^37,38^ combined with growth theory^38^ and references therein, together with innovation spread/diffusion^39^. Thus, our work directly relates to the collective dynamics of opinion formation for acceptances of innovations^40^) developed in the agent-based modelling and catalyst-based competitive survival model context. We dealt with two types of the non-equilibrium (irreversible) competitive game of life: (i) agent-based catalytic game of life, where the catalyst may be the effectiveness of state intervention, and (ii) agent-based autocatalytic game of life. The catalyst enters this game indirectly through the company’s technological level(fitness), while the descendant (created by a pair of agents) is the autocatalyst. This can be related by analogy to critical phenomena in catalytic and autocatalytic irreversible chemical reactions ^41,42^.

Our results show critical divergence of variance along the critical manifold associated with dysregulation of the agent’s system at critical levels of the catalyst. In the particular case of the market scenario, this warns against improper state interventions, especially for the market states in the multi-spectral critical points. From the point of view of econophysics, the various multi-spectral critical points represent the macrostates of the system with unlimited risk because then we deal with divergent fluctuations and susceptibilities in variables $q, \lambda$, and *z*. In effect, egalitarian state intervention either does little if it is below the critical thresholds even for a relatively large state intervention level, *q*, or it introduces a rapid increase in fluctuations and hence uncertainty and risk in the market when it passes the multi-spectral critical threshold.

Our work has universal hallmarks because it bridges and extends different branches of the science of complexity. Notably, these include the rapidly developing innovation diffusion/spreading discipline, the under-researched area of dissemination of innovative technologies in the market of competing companies and the potentially meaningful applications to chemical and biological reaction systems.

## Methodology, model, and method

The agent-based model used in this work can take multiple variants and configurations^36,43^ yielding itself to adaptation to the conditions imposed by a given market situation. We describe a variant of the model in which we observed multi-spectral criticality, not identified in the previous variants we investigated. Below, we only cover the elements of the model required for interpreting the results obtained. Detailed complementary information is provided in the Supplementary Information accompanying this work.

### Inspiration from company market

While the agent model can have many interpretations and applications, our motivation came from agent-based modelling of the competing companies’ markets in the presence of state intervention^36,43^. We are studying the market of competing companies as a primary market. In particular, the stock exchange would not exist without the market for competing companies/agents struggling to survive, which determine the dynamics/evolution of the stock exchange. State intervention in competing companies’ markets can tremendously impact the economy, mainly through technological growth. Understanding of the role of state interventionism in the free market economy is still in its infancy, although this problem is as old as the free market itself^44–46^.

Therefore, we introduce the following correspondences:

(i) agent—company
(ii) system—market
(iii) descendant—spin-off
(iv) death—bankruptcy
(v) fitness—technological level
(vi) rank—share
(vii) catalyst/catalysis—state intervention

which we use in the following as: ’company-based(agent-based).’

### Model substrate

We set up the model as follows. First, a two-dimensional regular lattice network of size $L \times L$ is created (where *L* is the linear size of the lattice). Each site *i* of the lattice represents a discrete location where a company(agent) can, but does not have to, reside. A pair of time-dependent functions describe the local state of the occupied site *i*:

1. The first function defines the company’s(agent’s) technology level(fitness), $A_i(t)$ at site *i*.
2. The second function represents the company market share(agent rank in the system) $\omega _i(t)$ at this site.

The company’s market share corresponds to the agent’s instantaneous rank in the agent community system formulated in the ABM language. At the same time, agent’s fitness represents its temporary ability to survive in that community, just as the company’s technological level assesses its temporary ability to survive on the market. Thus, the time-dependent microstate of the occupied lattice site is defined by the functions’ pair $(A_i(t),\omega _i(t))$, $i=1,2,\ldots ,N(t)$, which are the endogenous micro characteristics; *N*(*t*) is the endogenous macro characteristics specifying the number of agents present on the lattice at time *t*. An unpopulated lattice site is in the microstate of (0, 0).

Upon initialisation, the lattice sites are populated with probability $c_0$ subject to the condition of single occupancy per site. We chose $c_0=0.8$, meaning that, on average, the lattice starts with 80% of sites occupied. This particular choice allows us to compare our results with previous work^35,43^ (for $q\rightarrow 0$). Each occupied site is also assigned the technology level(fitness) $A_i(0)$ (drawn from a uniform distribution) and assigned equal shares $\omega _i(0)=\frac{1}{c_0L^2}$ to ensure egalitarian initial condition. The share(rank) $\omega _i(t)$ describes what percentage of the market(system) a given company(agent) at lattice site *i* holds at any time *t*. However, the leader defines an exogenous field; therefore, it is not included in the occupancy count. The normalisation condition,

17 $$\begin{aligned} \sum _{i=1}^{N(t)} \omega _i(t) = 1. \end{aligned}$$

holds for any given time *t*. Only *N*(*t*) lattice sites are populated at time *t*; the remaining $L^2-N(t)$ sites are empty.

### Model variables

Three stochastic variables $(q, \eta , \lambda )$ fully describe our model as follows.

**Figure 12:**
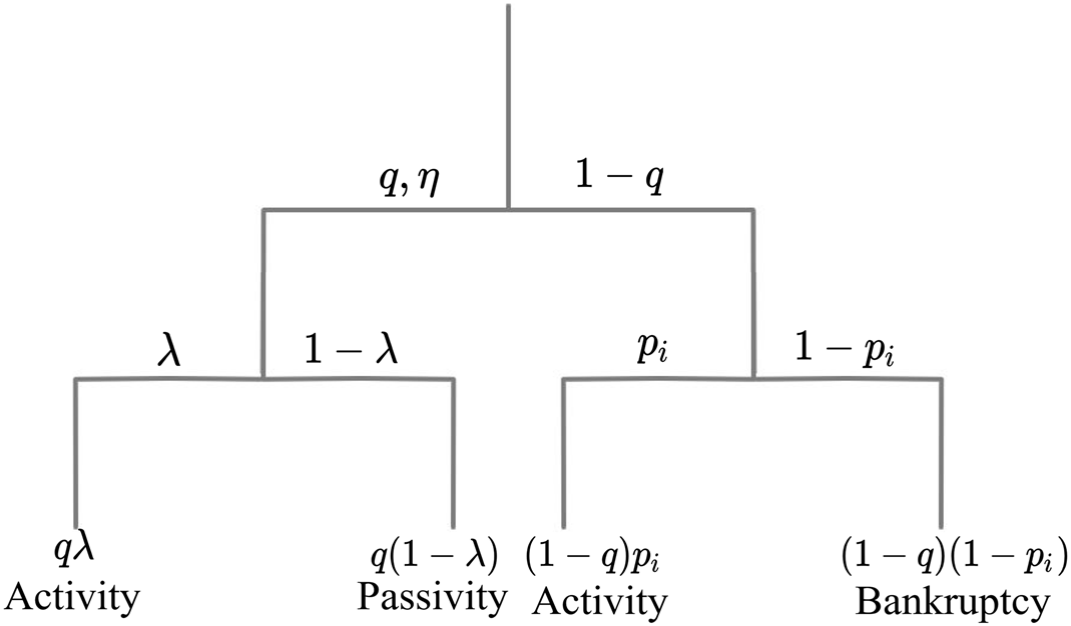
The hierarchical binary tree of life illustrates the functioning of our reference egalitarian variant of the model for any occupied lattice site. The right branch corresponds to no intervention with probability $1-q$, branching into Activity or Bankruptcy (death) according to the company’s(agent’s) survival probability $p_i$ given by Eq. (24) below. The left branch of the diagram represents the case of intervention with probability *q*, branching into Activity or Passivity according to the probability of the company’s(agent’s) activity probability $\lambda$. Option Activity is implemented differently on both branches, as seen in the example of Eqs. (20) and (21), which define the company’s technological growth. For the right branch, both efficiency and agent’s activity are assumed to equal 1, which compensates for the lack of state interventionism.

1. The first exogenous variable is the normalised level of state intervention(catalysis), $0\le q\le 1$, which describes how likely a company(agent) will receive support to shield it from bankruptcy(death).
2. The second variable, associated with both exogenous and endogenous properties of the model, is a normalised intervention(catalysis) efficiency, $0\le \eta \le 1$, which controls how much the technological level(fitness) of a company(agent) is expected to increase in a time unit upon receiving the state’s intervention(catalysis).
3. An important endogenous variable we consider is the normalised company(agent) activity, $0\le \lambda \le 1$, which is conditioned by successful state intervention (see the diagram in Fig. 12). This activity controls the time when the intervention(catalysis) starts, because after a successful intervention, the company needs some time to absorb this government support and reorganise accordingly.

Suppose the intervention(catalysis) did not occur with probability $1-q$ (see the right bifurcated branch of the binary tree of life in Fig. 12). In such a case, the company(agent) survives in the MCS at the *i*-th lattice site with probability $p_i$, being active in the same MCS (or having $\lambda =1$). The combined probability of the company’s(agent’s) survival on this branch equals $(1-q)p_i$. Thus, the total probability (i.e., on both branches) of the company’s(agent’s) survival in a given MCS equals $q+(1-q)p_i$. Otherwise, the company(agent) goes bankrupt(dies) with probability $(1-q)(1-p_i)$. Normalisation requires the probability that a company will go bankrupt(die) or survive to equal 1. In our variant of the model, the decision on state intervention (catalysis) is made before the survival probability of the company(agent) at the $i^{th}$ lattice site, $p_i(t)$, is calculated (see the binary tree in Fig. 12).

We consider an egalitarian approach, i.e., support through state intervention(catalysis) is random and independent of the company’s(agent’s) condition. This is applicable when the company’s(agent’s) state cannot be directly assessed. This type of intervention(catalysis) may occur, e.g., in a sudden economic or biological or ecological system collapse when the exogenous governing body does not have enough time to select companies(agents) in need. In this case, intervention(catalysis) follows the well-known principle of Steinhaus’ snapshot observations^47^ (see Ref.^43^ for the preferential rule case).

**Figure 13:**
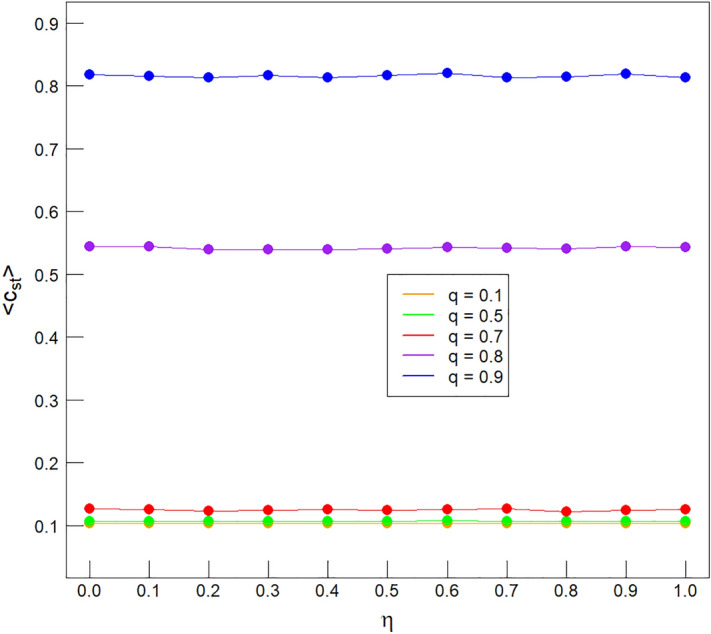
Example relationships of the mean population $\langle c_{st}\rangle ~$ vs. $\eta ~$ for five typical *q* values at fixed typical $\lambda =0.9$. Such a choice of these values $\lambda , q,\eta ~$ covers both phases—the bottom three curves belong to LP and the top two to HP. Technical note: filled circles indicate the simulation results and thin solid lines have been drawn between them to guide eye for interpolation. There is a clear dependence of $\langle c_{st}\rangle ~$ on *q* which was to be expected in the light of Fig. 1 and only a negligible, residual dependence on $\eta ~$—the catalyst level.

Our results depend weakly on intervention (catalysis) efficiency—i.e. variable $\eta ~$ (see Fig. 13). Thus we fix $\eta =0.5$ for simulations and do not interpret dependence of the results on this variable. Indeed, this dimensionality reduction allows us to represent the phase diagram embedded in a three- and not four-dimensional space in Fig. 1.

### Model evolution

The market(system) starts to evolve from an initial average technology level(fitness) $\overline{A(t=0)}$, which is set to $\overline{A(t=0)} = 0.5$. Random initial technology level(fitness) drawn from a uniform distribution $\mathscr {U}(0,1)$ over the segment $\langle 0,1\rangle$ ensures just that. We assume that $A_i(t=0)\le F(t=0),~i=1,2,\ldots , N(t=0)$, for self-consistency of the system as implied by the definition of the leader. At any given time *t* for a given realisation of the Monte Carlo simulation, the system is characterised by the average technology level(fitness) $\overline{A(t)}$, which is the average over the system of all the individual technology levels(fitnesses) weighed by the respective shares(ranks) for each *i*.

18 $$\begin{aligned} \overline{A(t)} = \sum _{i=1}^{N(t)} \omega _i(t) A_i(t). \end{aligned}$$

The model assumes that the progression of technology depends on the (global) technological growth rate of the leader. Companies in different countries can copy the leader’s solutions to increase technology. It constitutes the technology diffusion stimulated by the outer fields. The leader’s technology is assumed to grow according to Eq. (19) below, following Moore’s macroeconomic law of exponential growth^48^. The parameter $\sigma$ is the growth rate at which the leader’s technology, *F*(*t*), increases. So, we have,

19 $$\begin{aligned} F(t) = F(0)e^{\sigma t}, \end{aligned}$$

where we assume  $F(0)=1$ for further calculations for standardisation reason.

### Mutual interaction and fitness (technological level)

An important assumption of our model is that, apart from interventionism, the technological level is a decisive factor in companies’ survival probability and success. This follows a similar class of models^33–36^.

Let us first assume that there is no other company in the nearest vicinity of a given company located in $i^{th}$ lattice site. It may result from a fluctuation or when the concentration of agents on the lattice $c_ {st}\le 0.5~$. By $c_{st}$, we denote the concentration of agents on the lattice in the quasi-stationary macrostate of the system. Then, we can propose the linear first-order recursive equation describing the local (discrete) stochastic dynamics of the level of technology with multiplicative noise in the form:

20 $$\begin{aligned} A_i(t)-A_i(t-1)=r \gamma [F(t-1) - A_i(t-1)]\ge 0, \end{aligned}$$

where

21 $$\begin{aligned} \gamma = \left\{ \begin{array}{cc} \eta , & r<q \\ 1, & q\le r\le 1. \end{array} \right. \end{aligned}$$

Here, $t\ge 1$ (we measure it in MCS/site units), and $A_i$ represents the time-dependent company’s technology level in the $i^{th}$ lattice site, while *F* is the leader’s time-dependent technology level. The situation of the system corresponding to the left branch of the binary tree of life (shown in Fig. 12), i.e., the successful intervention consisting of drawing a random number $r<q$, we describe by the upper branch in Eq. (21). The lower branch in Eq. (21) refers to the right branch of the binary tree of life of a subbranch of ‘Activity’ (not ‘Bankruptcy’). Thus, the increase in the level of technology in Eq. (20) depends on the distance between the company’s level of technology, $A_i$, and the leader’s technological level(fitness), *F*. The greater this distance, the more significant the increase in the company’s technological level. We emphasise, that the influence of the variable *q* is through the range of values of the random number *r* defined above.

Let us assume, that there is another company *j* near a given company *i* (present in the lattice site). Then, one of the following two cases occurs:

(i) with a probability of *b* the company *i* acquires a company *j* by merging the companies, or
(ii) with the probability of $1-b$, both companies create a descendant (spin-off) in the first or second coordination zone of *i* site, provided that the empty lattice site locates in these zones. Therefore, here we are dealing with triadic dependence/interaction.

The equation of local dynamics related to the situation described in item (i) can be presented for the technological levels of companies as follows,

22 $$\begin{aligned} A_i(t)=max[A_i(t-1),A_j(t-1)]. \end{aligned}$$

On the other hand, the analogous equation for the case of item (ii) takes the form,

23 $$\begin{aligned} A_k(t)=max[A_i(t-1),A_j(t-1)], \end{aligned}$$

where *k* indexes the lattice site of the vacancy in the first or second coordination zones of lattice site *i*.

### Probability of company’s(agent’s) survival on lattice site

The time-dependent probability of a company’s(agent’s) survival within a single MCS/site given by Eq. (24) below is essential for defining the local dynamics. Its simple form was first introduced as the Ausloos–Clippe–Pekalski (ACP) model^33,49,50^—the ACP model uses only the third line of the extended model definition (24). The original APC model leads to the bankruptcy(death) of companies(agents) too quickly^35^, while our extended model (24) no longer has this disadvantage.

We focus on long-term macrostates of the system(market) where the technological level(fitness) $\overline{A(t)}\ge F(t=0)$, i.e., the companies’ technological levels weighted by their market shares(ranks) at time *t* (counted in Monte Carlo steps per site, MCSs/site); (for further details see Supplementary Information.

A situation in which the company did not receive government support is considered, i.e., the company is on the right branch of the diagram shown in Fig. 12. We now introduce the definition of $p_i(t)$ probability of the company’s survival in lattice site *i* in the time interval $[t,t+1]$. We quote this definition slightly more generally and modify the corresponding definition in Ref.^35^ For the randomly selected index value $1\le i\le L^2$ (where *L* is the linear size of the lattice), we can write,

24 $$\begin{aligned} p_i(t) = \left\{ \begin{array}{ll} e^{-sG_i(t)}, & \hspace{-1mm}\text {for}~~\overline{A(t)}< F(0) ~{\text {and}}~ G_i(t)\ge 0, \\ 1, & \hspace{-1mm}\text {for}~~ \overline{A(t)}< F(0)~{\text {and}}~G_i(t)< 0, \\ e^{-sH_i(t)}, & \hspace{-1mm}\text {for}~~\overline{A(t)}\ge F(0), \end{array}\right. \end{aligned}$$

where $p_i(t)$ vanishes for empty lattice site *i* and $p_i(t)=1$ whenever the number of agents on the network reaches the set minimum value $N_{min}$. This is the lower boundary condition protecting the market from collapse. Moreover, in one MCS/site (i.e., $L^2$ MCSs), each lattice site is, on average, drawn once because all of them have the same probability of appearance $1/L^2$. In the MC algorithm, we consider, as usual, two-time scales: (i) Monte Carlo step (MCS) or a single MC draw and (ii) Monte Carlo step per lattice site (MCS/site). Concerning time scale (i), the time $t_{MCS}$ is simply the number of draws, and (in the case of sequential selection of lattice sites and not randomising them) we have the relation $t_{MCS}=L^2~t+i$, where $0\le i\le L^2$. In the case of draws, the order which appears is random.

By definition, $G_i(t)=\frac{\overline{A(t)}}{F(0)}F(t) - A_i(t)$, where $A_i(t)$ is the technology level(fitness) of the company(agent) at a site *i* at time *t*; analogously, by definition $H_i(t)=F(t) - A_i (t)$. Note, that if $G_i(t)<0$, then the company(agent) located at site *i* has such a significant/large level of technology (fitness) $A_i(t)$ at time *t* that it will undoubtedly survive at this lattice site until the following MCS/site. Obviously, (for long times) $G_i(t)\ge 0$, but in the definition of $p_i(t)$ we insert a quantity with a smaller value, i.e. $H_i(t)$. Notably, $H_i(t)$ is always non-negative because the technological level(fitness) of a given company(agent) located at arbitrary site *i* never exceeds that of the leader; we assume that the leader remains the same during the system’s evolution. Furthermore, as defined by *F*(*t*), we have $A_i(0)<F(0)$ for each lattice site *i* occupied by the company(agent).

Parameter *s*, constant/uniform in time and space, describes selection pressure^49^. If $s=0$, the companies(agents) never go bankrupt(die). When the *s* parameter increases, the probability of survival of a given company(agent) decreases, i.e., high *s* means that less developed companies(agents) have a higher risk of bankruptcy(death). In short, higher *s* leads to faster fitness(technological level) growth of the system because the system gets rid of technologically weak companies(agents with low fitness). The egalitarian variant requires small values of *s* (here, $s=1$ was used) in order significantly to increase the chances of survival of weak companies(agents with low fitness), i.e., *s* is a parameter which controls the equalisation of companies’(agents’) chances of survival in the system. Hence, we made the survival probability of the company(agent) at a given lattice site dependent on the distance between its technological level(fitness) and the technological level(fitness) of the leader. However, in the fourth line of Eq. (24), we added a blocking boundary condition securing survival on the market for a minimum number of companies(agents), $N_{min}$.

Summarising, we have shown the way from the control variables, $(\lambda ,q, \eta )$, of the model through the time-dependent technology levels(fitnesses), $A_i(t)$, to the basic survival probabilities of companies(agents), $p_i(t)$, on the lattice site *i*. It is the technological level(fitness) which controls the local dynamics/kinetics of the company(agent)—the company(agent) tries to achieve the technological level(fitness) of a leader, competing with other companies(agents) for survival in the market—therefore, except for the leader, the company’s(agent’s) technological levels(fitnesses) are the central endogenous characteristics of our model.

## Data Availability

The datasets generated and/or analysed during the current study are available from one of us (M.Ch.) upon reasonable request.
